# Dual immunomodulatory and antileishmanial potential of TLR7/8 agonists against *Leishmania donovani*

**DOI:** 10.1128/iai.00323-25

**Published:** 2025-10-20

**Authors:** Shivani Thakur, Deepender Kaushik, Kushvinder Kumar, Sandeep Kaur, Ravinder Kumar, Deepak B. Salunke, Sukhbir Kaur

**Affiliations:** 1 Parasitology Laboratory, Department of Zoology, Panjab University 29750, Chandigarh, India; 2 Department of Chemistry and Centre of Advanced Studies in Chemistry, Panjab University 29750, Chandigarh, India; 3 National Interdisciplinary Centre of Vaccine, Immunotherapeutics and Antimicrobials (NICOVIA), Panjab University 29750, Chandigarh, India; 4 Department of Medicinal Chemistry, National Institute of Pharmaceutical Education and Research, Mohali, Punjab, India; Tulane University, New Orleans, Louisiana, USA

**Keywords:** TLR7/8 agonists, *Leishmania donovani*, nitric oxide, ROS, PI assay, cytotoxicity, host-directed therapy, immunomodulation, visceral leishmanias

## Abstract

Visceral leishmaniasis (VL), caused by *Leishmania donovani*, is a neglected tropical disease with limited therapeutic options and increasing drug resistance. This study investigates the immunological mechanisms and antiparasitic efficacy of imidazoquinoline-based Toll-like receptor 7/8 (TLR7/8) agonists as host-directed agents in an *in vitro* VL model. Using RAW 264.7 macrophages and *L. donovani* promastigotes and amastigotes, we examined macrophage activation, nitric oxide (NO) induction, and cell cycle disruption in parasites. The lead compounds (5 and 10) significantly enhanced NO production in macrophages, both in unstimulated and LPS-stimulated conditions, indicating robust innate immune activation. Additionally, parasite-derived reactive oxygen species (ROS) levels were markedly elevated, suggesting oxidative stress as a mechanism of direct leishmanicidal action. Flow cytometric analysis revealed G_0_/G_1_ arrest in treated promastigotes, further supporting interference with parasite proliferation. Importantly, these compounds exhibited low cytotoxicity toward host cells and favorable selectivity indices. Notably, this is the first *in vitro* study to comprehensively demonstrate the ability of TLR7/8 agonists to exert direct parasiticidal effects along with immune modulation in the context of VL. The results underscore the potential of TLR-targeted immunomodulation to enhance host defense mechanisms against intracellular protozoan infections and contribute to the development of novel immunopharmacological interventions for VL.

## INTRODUCTION

Leishmaniasis is among one of the most neglected tropical diseases caused by the intracellular protozoan of genus *Leishmania*. The disease is prevalent predominantly in tropical and subtropical regions, affecting more than 12 million people worldwide. The disease manifests mainly in three forms: cutaneous leishmaniasis (CL), mucocutaneous leishmaniasis, and visceral leishmaniasis (VL) (1). VL is the most severe and potentially fatal form caused mainly by *Leishmania donovani* and *Leishmania infantum*, affecting internal organs such as the spleen, liver, and bone marrow, resulting in fatalities if left untreated (2). Regardless of extensive research over years, adequate control and elimination of VL remain difficult, primarily due to lack of a potent vaccine and limitations of existing treatment options. Current treatment options, such as amphotericin B (AmB), miltefosine (MF), and pentavalent antimonials, face problems of toxicity, drug resistance, high cost, and longer duration of treatment. Therefore, immediate exploration of some novel therapeutic approaches that do not only target parasites but also modulate the host immune response leading to parasite elimination is required (3).

In infected hosts, *Leishmania* parasites are obligate intracellular microorganisms that reside and replicate within the phagolysosomal compartment of host macrophages, a niche that enables them to evade many extracellular immune defenses. Macrophages are professional phagocytes, serving as the primary host cells for *Leishmania* and are central to innate immunity, functioning as both a physical habitat for the parasite and a pivotal effector of host defense through antigen presentation, production of reactive oxygen and nitrogen species, and orchestration of inflammatory responses (4). Upon exposure to infection, promastigotes are phagocytosed by macrophages and transform into amastigotes. To successfully evade the host immune system, *Leishmania* has evolved an advanced mechanism to weaken the immune responses, such as suppression of reactive oxygen species (ROS) and nitric oxide (NO) production, as well as modulating inflammatory cytokine response (5). The protective immune response against infection is T cell dependent, with generation of a strong Th1-type response essential for the elimination of parasite. Once activated, the macrophages produce ROS, NO, and pro-inflammatory cytokines, which collectively contribute to selectively killing the parasite. In VL, sometimes the immune dysfunction and deactivation of macrophages lead to the inability of host cells in clearing the parasites. This highlights the role of macrophages as primary host cells and makes them a prime target for host-directed therapies (6). This specialized adaptation not only fascinates macrophage immunologists but also drives the concept of parasite clearance via host-directed immunological mechanisms (7). Recognition of pathogen-associated molecular patterns (PAMPs) is primarily done by Toll-like receptors (TLRs), which are the key receptors of the host immune system, leading to initiation and activation of innate immune response. Among the 13 TLRs, TLR7 and TLR8 are predominantly located within the endosomal compartments and play an important role in recognizing the single-stranded RNA from different pathogens, including *Leishmania*. Various research investigations have depicted that certain synthetic Toll-like receptor 7/8 (TLR7/8) agonists can effectively activate the macrophages and other immune cells, leading to the generation of pro-inflammatory cytokines like tumor necrosis factor alpha (TNF-α), interleukin (IL)-6, and IL-12, contributing to Th1 immune response (8). Synthetic TLR7/8 agonists have shown promise in modulating immunity and enhancing macrophage activation. Preclinical studies in CL models have demonstrated that imidazoquinoline-based agonists such as imiquimod and resiquimod can activate immune cells and promote parasite clearance when used alone or in combination with antigens (9, 10). Importantly, imiquimod has also been used clinically in CL, where topical application in combination with antimonials improved lesion healing and parasite clearance (11). However, these studies have largely focused on topical or localized immune responses in CL, and similar strategies remain largely unexplored in systemic infections like VL.

Although TLR7/8 agonists have been studied for their immunostimulatory effects in CL and some *in vitro* antileishmanial activity (7), their dual potential of simultaneously activating host immunity and directly targeting *L. donovani* remains largely unexplored in systemic VL. In particular, a comprehensive evaluation of their effects against both intracellular amastigote stage, alongside cytotoxicity profiling in immune and non-immune cells, is lacking.

It is important to distinguish that immunomodulatory activity refers to the ability of TLR7/8 agonists to activate host immune cells, particularly macrophages, leading to the production of NO, ROS, and pro-inflammatory cytokines. This activation enhances the host’s immune response, indirectly contributing to parasite killing (9). In contrast, antileishmanial activity refers to the direct effect of these compounds on the parasite itself, independent of host immune activation. Their amphipathic, planar aromatic structures and lipophilicity allow passive diffusion into parasites, where they can disrupt mitochondrial function, induce ROS, cause cell cycle arrest, and trigger apoptosis-like pathways. Previous reports, including imiquimod combinations, have confirmed receptor-independent, direct killing of promastigotes and amastigotes (12, 13).

An extensive evaluation of TLR7/8 agonists requires assessment of their antileishmanial efficacy against both promastigote and amastigote stages (Half-maximal Inhibitory Concentration [IC_50_]), as well as their cytotoxicity (50% cytotoxic concentration [CC_50_]) evaluation in both immune and non-immune cells. In addition to assessing cytotoxicity on RAW 264.7 macrophages, we included HeLa cells, a non-phagocytic human epithelial cell line, to evaluate general safety and off-target effects. Similar approaches have been used in previous studies, such as bis-(4-hydroxycoumarin-3-yl) methane derivatives and phthalimido-thiazole derivatives, which were tested in multiple cell lines, including HeLa, to ensure comprehensive safety profiling (14, 15). Evaluating CC_50_ on both RAW 264.7 macrophages and HeLa cells provides better understanding of selective toxicity, and comparison with IC_50_ values allows calculation of selectivity index (SI), an important indicator of therapeutic potential ensuring selective parasite killing with minimal host cell toxicity (7). Antipromastigote assay determines that compounds have a direct effect against the extracellular infective stage, and antiamastigote assay determines their capacity to target the intracellular stage, which is responsible for pathogenesis. Assessing ROS levels in promastigotes after treatment with TLR7/8 active compounds plays an important role in understanding their direct effect on the parasites. Enhanced ROS levels can lead to oxidative damage of proteins, DNA, and lipids, potentially arresting the promastigotes at a specific stage of the cell cycle. Thus, analyzing cell cycle stage of promastigotes by propidium iodide (PI), along with ROS analysis, is important to check whether the compound can impair *Leishmania* proliferation and survival of it (9). Estimation of NO levels after treatment with compounds is crucial to evaluate their ability to enhance macrophage antiparasitic responses against *Leishmania*. NO is one of the key effector molecules in intracellular killing of parasites by inducing nitrosative stress (16).

Considering the central role of macrophages in both pathogenesis and clearance of infection and the potential of synthetic TLR7/8 agonists as both antiparasitic and immunomodulatory agents, the present study builds on previous work in CL to provide the first systematic evaluation of a panel of 11 well-characterized imidazoquinoline-based TLR7/8 agonists against VL caused by *L. donovani*. This study explicitly highlights the novelty of using TLR7/8 agonists for their dual activity, where IC_50_ values against intracellular amastigotes and the NO-generating capacity of macrophages after treatment indicate immunomodulatory potential, while ROS induction, cell cycle arrest, and IC_50_ against promastigotes reflect direct parasiticidal effects. Cytotoxicity profiling in both RAW 264.7 macrophages and HeLa cells allowed calculation of SI, ensuring therapeutic selectivity. By integrating direct parasite-targeted activity with host-directed immune activation, this work establishes a translational framework for developing TLR7/8-based therapeutics for VL.

## MATERIALS AND METHODS

### TLR7/8 agonists

Imidazoquinoline-based TLR7/8 agonists were selected from the recent structure–activity relationship study we performed to identify potent vaccine adjuvant. Synthesis of the selected compounds was achieved following the methods reported. The structures of the compounds were confirmed by spectroscopic analysis, and the purity of all the analogs was assessed by high-performance liquid chromatography. All the compounds with purity of ≥95% were used for biological evaluation (17–19).

### Culture of promastigotes

Promastigotes of Indian strain of *L. donovani* MHOM/IN/80/DD8 were maintained in Medium 199 (Sigma-Aldrich, USA) at an ambient temperature of 22°C ± 1°C. M199 was added with 0.2% sodium bicarbonate and 10% heat-inactivated fetal bovine serum (FBS; Biowest, France) + antibiotics (gentamycin and streptomycin) by serial subcultures after every 48–72 hours by transferring 0.5–1.0 mL of culture supernatant in fresh medium and kept in a biological oxygen demand (BOD) incubator (20).

### Culture of RAW 264.7 macrophages and HeLa cells

To evaluate the cytotoxicity and antiamastigote assay, RAW 264.7 macrophages were cultured in Dulbecco’s modified Eagle medium (HiMedia, India), 10% heat-inactivated fetal bovine serum (FBS, Biowest) and cultured at 37°C (5% CO_2_). The macrophage cell growth was evaluated daily, and the cells were subcultured into a new flask containing fresh cell culture medium once they reached about 80%–90% confluency.

HeLa cells (human cervical epithelial cell line) were grown in RPMI-1640 (HiMedia) with 10% FBS and cultured at 37°C (5% CO_2_). The cells were evaluated daily using an inverted microscope for any contamination and subcultured after reaching confluency (21).

### *In vitro* antipromastigote activity (IC_50_ assay)

Promastigotes (2 × 10^6^ /mL) were plated with different doses of TLR7/8 agonists (2–64 µg/mL) in 48-well tissue culture plates in triplicate. AmB (0.1–2.0 μg/mL, Sigma-Aldrich), MF (TCI, Japan), and Resiquimod (known dual TLR7/8 agonist) (TCI) were taken as reference drugs. After 48 hours of incubation, parasites were stained with trypan blue (Sigma-Aldrich) to differentiate between live and dead promastigotes under light microscope. The percentage growth inhibition was calculated using the following formulas:


$$Percentage\;viability=\frac{No.\;of\;live\;cells\;in\;treated\;well}{No.\;of\;live\;cells\;in\;blank\;well}\times 100$$

Percentage growth inhibition=100 − percentage viability.

The IC_50_ values were calculated using SPSS software, version 25 (22).

### Assessment of antiamastigote activity

To determine the antileishmanial efficacy, RAW macrophages were parasitized with promastigotes and treated with TLR7/8 agonists, MF and Resiquimod at concentrations of 2, 4, 8, 16, 32, and 64 µg/mL and AmB at 0.1–2.0 µg/mL (10, 12). Macrophages (2 × 10^4^/mL) were plated on round coverslips placed in 24-well plates for 24 hours in a CO_2_ incubator (5% CO_2_) at 37°C. The coverslips were washed thrice with serum-free medium to remove the non-adherent cells. The cells were then infected with promastigotes in stationary phase at a ratio of 1:10 (macrophage : *Leishmania*) for 24 hours. The cells were washed to remove the non-phagocytosed promastigotes. Infected macrophages were incubated with different concentrations of TLR7/8 agonists for 48 hours. After 48 hours, the coverslips were washed with phosphate-buffered saline (PBS), dried, fixed with chilled methanol, Giemsa-stained, and examined under a light microscope. A minimum of 200 macrophages were counted per coverslip to determine the number of amastigotes. The IC_50_ value was calculated using SPSS software, version 25 (21).

### Cytotoxicity of TLR7/8 agonists on RAW 264.7 macrophages and HeLa cell lines

The cytotoxicity studies were performed using an immune cell line (RAW 264.7 macrophage) and non-immune epithelial cell line (HeLa) by 3-(4,5-dimethylthiazol-2-yl)-2,5-diphenyltetrazolium bromide (MTT) assay. A total of 7,000–10,000 cells/well were supplemented with different concentrations (2–64 μg/mL) of TLR7/8 agonists and incubated for 48 hours. MTT (1 mg/mL) (Hi-Media) was added to each well and kept for 4 hours. The formazan crystals formed were dissolved in 100 µL of dimethyl ulfoxide (DMSO), and absorbance at 550 nm was recorded using a microreader. AmB, MF, and Resiquimod were used as reference drugs. The percentage of cytotoxicity was calculated as [(absorbance of treated well − absorbance of blank) / (absorbance of untreated well − absorbance of blank)]  × 100. The CC_50_ value was calculated with SPSS software. The SI of TLR7/8 agonists was obtained by calculating the ratio of CC_50_ (RAW 264.7 macrophage and HeLa cells) and IC_50_ (antipromastigote and antiamastigotes) (21, 23).

### Estimation of ROS in *Leishmania* promastigotes following treatment with test compounds

Induction of ROS by promastigotes after exposure to TLR7/8 agonists was done using a cell-permeable fluorescent dye H_2_DCFDA (Thermo Fisher). Briefly, 1 × 10^6^ parasites/mL were incubated with the IC_50_ value of TLR7/8 agonists, AmB, MF, and Resiquimod for 48 hours. After this given time period, parasites were harvested and washed thrice with PBS and resuspended in 10 µM H_2_DCFDA for 20 minutes in the dark at 37°C. The fluorescence was measured by using FACS Calibur flow cytometry (24).

### Estimation of NO production in RAW 264.7 macrophages after treatment with TLR7/8 agonists

RAW 264.7 macrophages (1 × 10^6^) were added to a sterile 96-well plate and incubated with the IC_50_ value of TLR7/8 agonists. After 24 hours, non-adherent cells were removed, and adherent cells were incubated with TLR7/8 agonist for 48 hours in the presence or absence of LPS (1 µg/mL) (Biosciences) to evaluate both unstimulated and LPS-stimulated macrophage responses. After 48 hours, the culture supernatant (100 µL) was collected and mixed with an equal volume of Griess reagent (1% sulfanilamide and 0.1% naphthlethylenediamine dihydrochloride in 2.5% phosphoric acid) (Sigma), and absorbance was measured in a microreader at 540 nm. Nitrite concentration was evaluated from a standard curve generated with sodium nitrite (25).

### Assessment of cell cycle progression in *Leishmania* promastigotes using propidium iodide staining

For analysis of the promastigote cell cycle, 2 × 10^6^ promastigotes/mL were briefly incubated with the IC_50_ of TLR7/8 agonists in a 24-well culture plate. After 48 hours, promastigotes were washed with PBS and fixed with 70% (vol/vol) chilled ethanol. After 1 hour of incubation, parasites were washed with PBS, and the pellet was incubated with 500 µL of DNase-free RNase (200 µg/mL) (Thermo Fisher) for 1 hour. Afterward, parasites were stained with PI dye (50 µg/mL), and the percentage of parasites in the sub-G_0_/G_1_ phase was analyzed using FACS Calibur flow cytometry (20).

## RESULTS

### *In vitro* antiproliferative activity of imidazoquinoline-based TLR7/8 agonists against *Leishmania donovani* promastigotes

**Fig 1:**
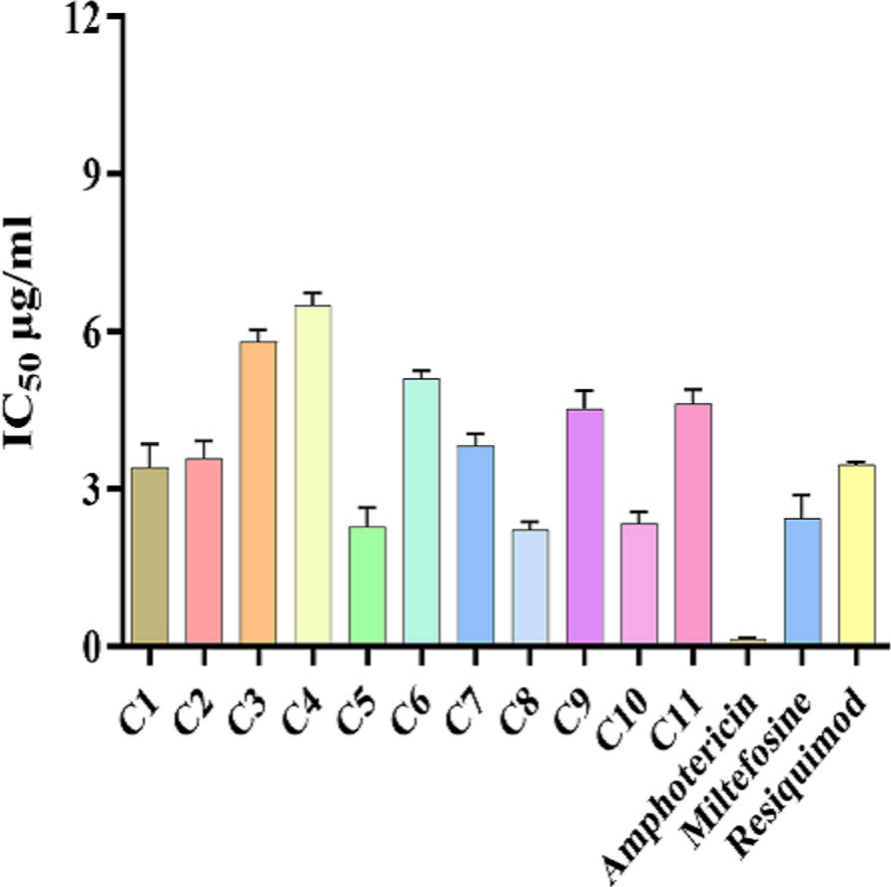
Inhibitory concentrations (IC₅₀) of imidazoquinoline-based TLR7/8 agonist against *L. donovani* promastigotes. Each bar represents the mean ± standard deviation of compounds (C1–C11) from three independent experiments performed in triplicate. Statistical significance was analyzed using one-way analysis of variance followed by Tukey’s multiple comparison test.

The treatment of promastigotes of *L. donovani* with the TLR7/8 agonists promptly reduced the growth of parasites (Fig. 1). The IC_50_ values ranged from 2.28 ± 0.62 µg/mL to 6.5 ± 0.4 µg/mL. Among the 11 tested derivatives, compounds 5 and 10 depicted the best antileishmanial activity with the least IC_50_, i.e., 2.28 ± 0.62 µg/mL and 2.33 ± 0.46 µg/mL. The IC_50_ values of standard antileishmanial drugs AmB, MF, and resiquimod were 0.13 ± 0.06 µg/mL, 2.43 ± 0.77 µg/mL, and 3.46 ± 0.1 µg/mL, respectively. While none of the test compounds exhibited greater potency than AmB, three derivatives (compound 1, compound 5, and compound 10) displayed comparable activity to MF. However, in comparison to Resiquimod, seven derivatives showed lower IC_50_ values. These results identify compounds 5 and 10 as promising candidates for further investigation.

### *In vitro* evaluation of intracellular *Leishmania donovani* amastigote clearance in RAW 264.7 macrophages

**Fig 2:**
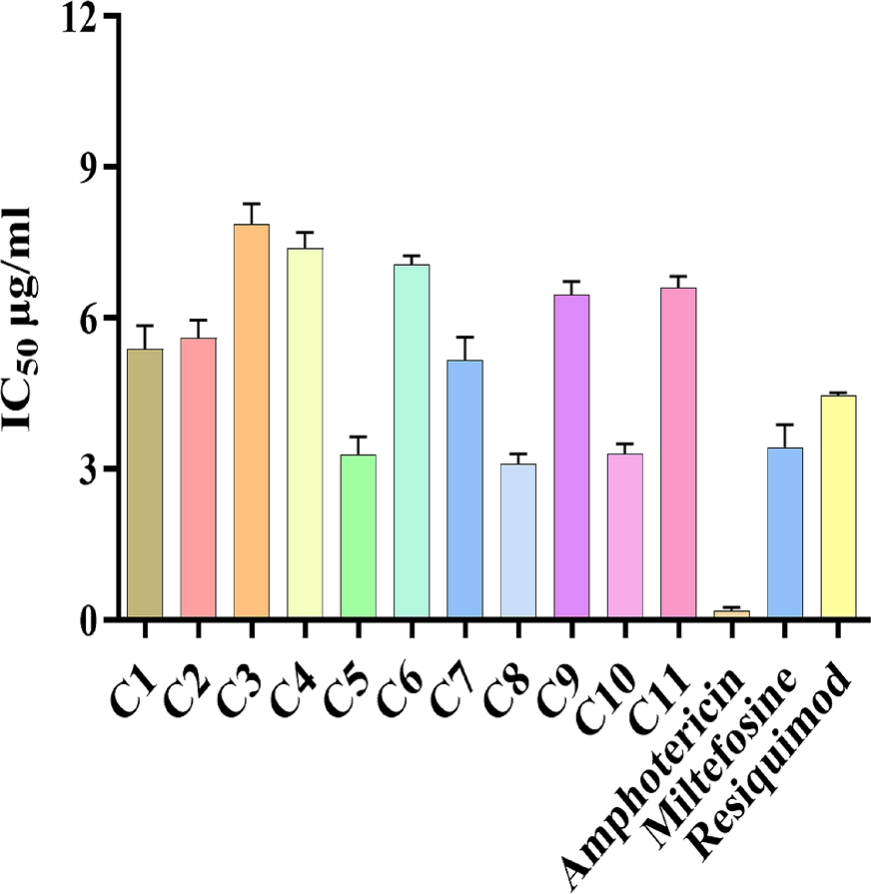
RAW 264.7 macrophages infected with *L. donovani* were tested against the TLR7/8 agonists. Each bar represents the mean ± standard deviation of compounds (C1–C11) from three independent experiments performed in triplicate. Statistical significance was analyzed using one-way analysis of variance followed by Tukey’s multiple comparison test.

**Fig 3:**
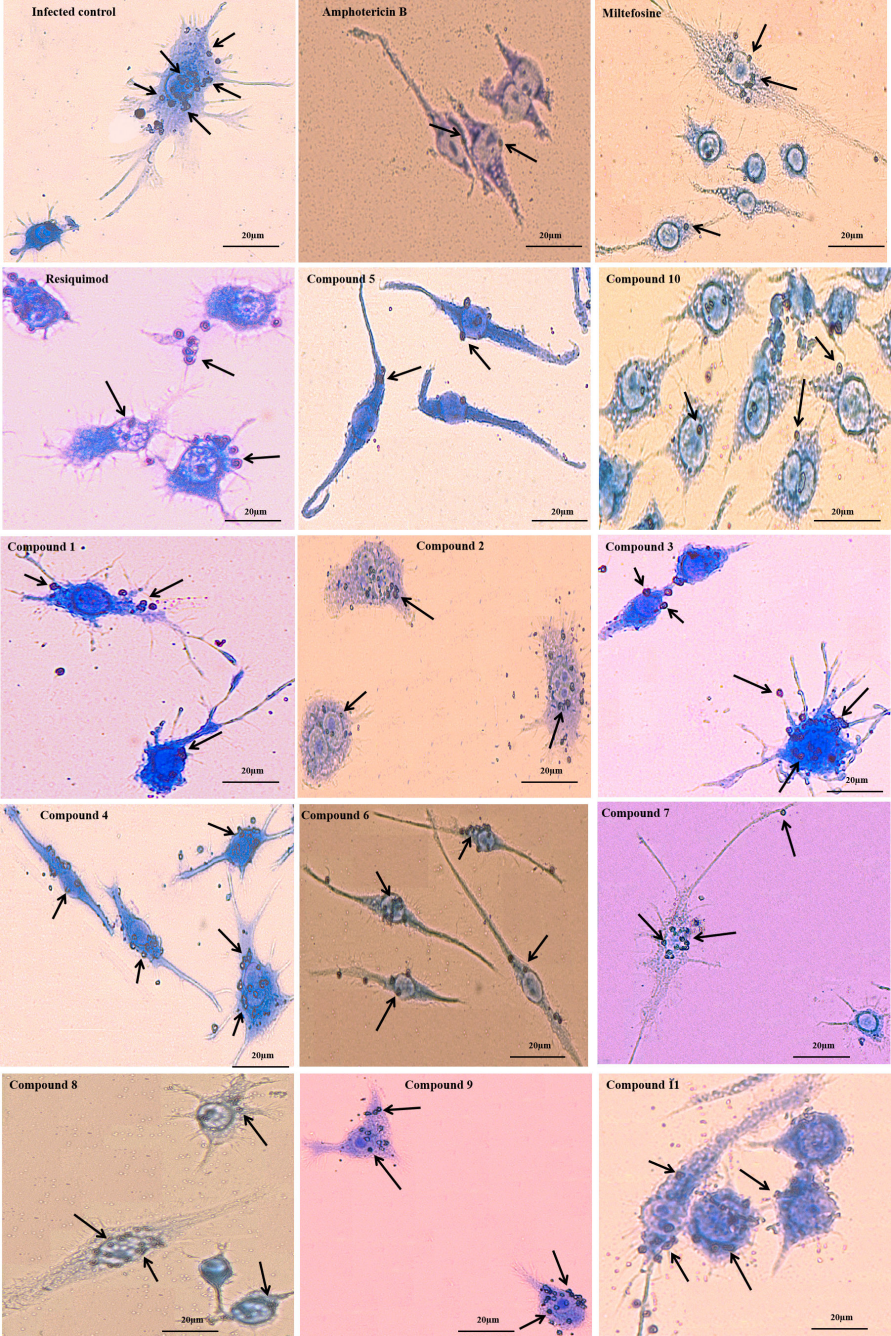
Giemsa-stained RAW 264.7 Macrophages showing intracellular amastigotes after treatment with control, standard drugs (AmB, MF, Resiquimod), and test compounds (1–11). Arrows indicate intracellular amastigotes.

To evaluate TLR7/8 agonist efficacy against intracellular amastigotes, RAW 264.7 macrophages were infected with *L. donovani* promastigotes and subsequently treated with the test compounds. The results depicted that all the 11 derivatives reduced the number of amastigotes (Fig. 2). Among all the derivatives, compounds 5 and 10 showed the least IC_50_ values, i.e., 3.29 ± 0.61 µg/mL and 3.0 ± 0.36 µg/mL. However, the IC_50_ values of remaining derivatives were compound 1 (5.4 ± 0.79 µg/mL), compound 2 (5.6 ± 0.62 µg/mL), compound 3 (7.86 ± 0.7 µg/mL), compound 4 (7.4 ± 0.52 µg/mL), compound 6 (7.06 ± 0.3 µg/mL), compound 7 (5.16 ± 0.79 µg/mL), compound 8 (3.1 ± 0.9 µg/mL), compound 9 (6.46 ± 0.47 µg/mL), and compound 11 (6.6 ± 0.4 µg/mL). When standard antileishmanial drugs AmB, MF, and Resiquimod were tested, IC_50_ values of 0.2 ± 0.1 µg/mL, 3.43 ± 0.77 µg/mL, and 4.46 ± 0.1 µg/mL were achieved. Notably, compounds 5 and 10 demonstrated antiamastigote activity superior to Resiquimod, underscoring their potential as lead candidates for further preclinical evaluation. To further substantiate the antiamastigote efficacy of the compounds, representative Giemsa-stained micrographs of *L. donovani*-infected RAW 264.7 macrophages following treatment with test derivatives and standard drugs are shown in Fig. 3.

### *In vitro* cytotoxic profiling of synthetic TLR7/8 agonists in HeLa cells

**Fig 4:**
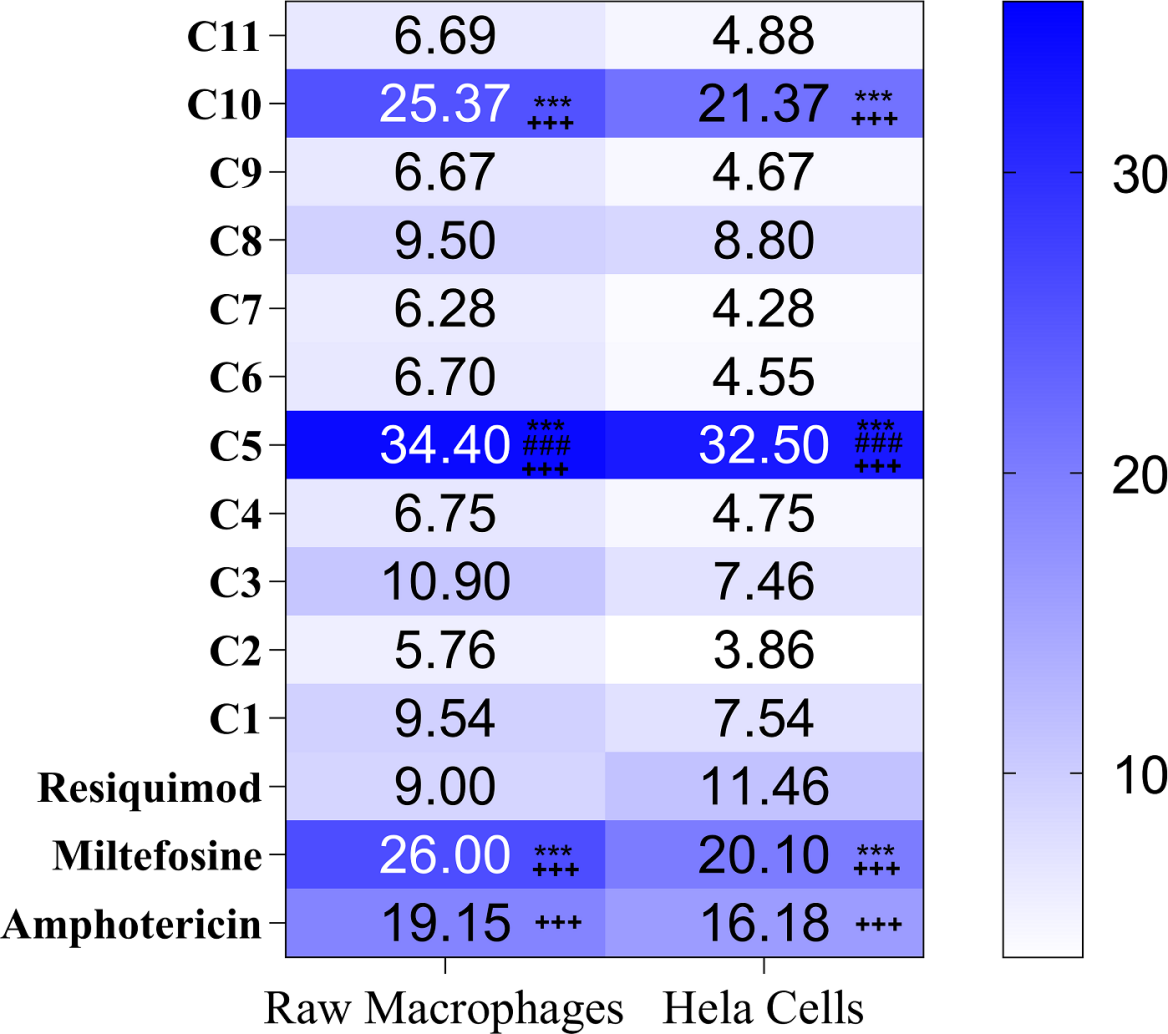
Heat map depiction of CC_50_ values for TLR7/8 agonists (C1–C11) in HeLa and RAW 264.7 cells. The color gradient indicates the cytotoxicity levels; darker shades depict higher CC_50_ values (lower toxicity), and lighter shades depict lower CC_50_ values (higher toxicity). Data represent mean CC_50_ values from three independent experiments (^+++^*P*<0.001, Resiquimod vs AmB, MF, compound 5, and compound 10; ***P<0.001, AmB vs MF, compound 5, and compound 10; ^###^*P* < 0.001, MF vs compound 5). The statistical significance was determined using one-way analysis of variance (Tukey’s multiple comparison test).

To evaluate host cell safety, the cytotoxic potential of imidazoquinoline-based TLR7/8 agonists was assessed in HeLa cells using the MTT assay. Cells were treated with a range of compound concentrations for 48 hours, and the CC_50_ values were calculated from three independent experiments. Among the tested derivatives, maximum CC_50_ value was observed for compound 5 (32.5 ± 0.5 µg/mL), which was significantly higher (*P* < 0.001) than all the compounds including standard AmB, MF, and Resiquimod. Compound 10 showed a CC_50_ value of 29.37 ± 1.6 µg/mL, which was significantly higher than most of the TLR/8 agonists, including Resiquimod (*P* < 0.001). The CC_50_ value of the remaining nine compounds ranged from 1.76 ± 0.43 µg/mL to 9.53 ± 0.41 µg/mL. Standard antileishmanial drugs AmB and MF showed CC_50_ values of 20.18 ± 0.72 µg/mL and 32.1 ± 0.94 µg/mL; Resiquimod showed a CC_50_ value of 9 ± 0.16 µg/mL (*P*  <  0.001) (Fig. 4). Remarkably, the significantly higher CC_50_ values of compounds 5 and 10 suggest an improved safety profile relative to the reference compound Resiquimod, warranting their further exploration as selective immunotherapeutic agents.

### Impact of TLR7/8-targeting imidazoquinolines on the viability of RAW macrophages

The cytotoxicity was evaluated in RAW macrophages, and cells were treated with increasing concentration of all derivatives (Fig. 4). Cells were exposed to increasing concentrations of each compound, and CC_50_ values were determined from three independent experiments, expressed as mean ± SD (Fig. 4). The CC_50_ value of compound 5 was 34.4 ± 0.5 µg/mL, and compound 10 was 30.37 ± 1.6 µg/mL, making them the least toxic, followed by compound 3 (10.9 ± 0.26 µg/mL) < compound 8 (9.50 ± 1.0 µg/mL) < compound 1 (9.54 ± 0.62 µg/mL) < compound 4 (6.75 ± 0.21 µg/mL) < compound 6 (6.70 ± 0.59 µg/mL) < compound 9 (6.67 ± 0.34 µg/mL) < compound 11 (6.69 ± 0.22 µg/mL) < compound 7 (6.28 ± 0.28 µg/mL) < compound 2 (5.76 ± 0.66 µg/mL). TLR7/8 agonist Resiquimod and standard antileishmanial drugs AmB and MF showed CC_50_ values of 7.46 ± 0.5 µg/mL (*P*  <  0.001), 19.15 ± 1 µg/mL, and 26.0 ± 1.0 µg/mL. Among all derivatives, compound 5 had CC_50_ values significantly higher than all the compounds, including AmB, MF, and Resiquimod (*P* < 0.001), and compound 10 had CC_50_ values significantly higher than all derivatives, AmB, and Resiquimod (*P* < 0.001).

When compared across cell lines, cytotoxic effects were generally more pronounced in HeLa epithelial cells than in RAW macrophages. This differential response likely reflects the intrinsic immune resilience of macrophages, which are evolutionarily adapted to tolerate inflammatory stimuli, unlike the more sensitive, non-immune HeLa cells. These findings underscore the selective tolerability of the lead compounds in immune-relevant cells, supporting their candidacy for further host-directed therapeutic development.

### Selectivity index of TLR7/8 agonists in HeLa and RAW 264.7 cells

The SI of TLR7/8 agonists was evaluated by calculating the ratio of CC_50_ (RAW 264.7 cells and HeLa cells) to IC_50_ (*L. donovani* promastigotes and amastigotes) (Table 1). The CC_50_ values of TLR7/8 agonists for HeLa cells ranged from 3.42 ± 0.5 µg/mL to 32.4 ± 0.52 µg/mL, and those for RAW macrophages ranged from 5.42 ± 0.5 µg/mL to 34.4 ± 0.52 µg/mL. The IC_50_ values of TLR7/8 agonists were also evaluated for both promastigotes and intracellular amastigotes of *L. donovani*, and the values were in the range of 2.28 ± 0.62 µg/mL to 6.5 ± 0.4 µg/mL for antipromastigotes and 3.29 ± 0.61 µg/mL to 7.4 ± 0.52 µg/mL for antiamastigote assay. A higher SI value depicts considerable selectivity of TLR7/8 agonists with regard to parasites and lower cytotoxicity to mammalian cells. In this study, two compounds showed the highest SI values: compound 5, 15.08 and 10.45 for antipromastigotes and antiamastigotes against RAW macrophages, and compound 10, 12.6 and 9.79 SI values for antipromastigotes and antiamastigotes against RAW macrophages. However, against the HeLa cell line, the SI values showed a minute decline in the values, i.e., 14.25 (antipromastigote) and 9.87 (antiamastigote) for compound 5 and 12.6 (antipromastigote) and 9.79 (antiamastigote) for compound 10. Both compounds exhibited SI values exceeding 10 in multiple assays, suggesting excellent therapeutic potential and highlighting their promise for further development as immunomodulatory therapeutic and prophylactic agents against visceral leishmaniasis.

**TABLE 1:** Selectivity index of TLR7/8 agonists on RAW macrophages and HeLa cell lines^*a*^

Compd.	Structure	TLL7 EC_50_	TLR8 EC_50_	IC_50_ (antipromastigote) (μg/mL)	IC_50_ (antiamastigote) (μg/mL)	RAW macrophage			HeLa cell		
						CC_50_ (μg/mL)	SI (antipromastigote)	SI (antiamastigote)	CC_50_ (μg/mL)	SI (antipromastigote)	SI (antiamastigote)
1	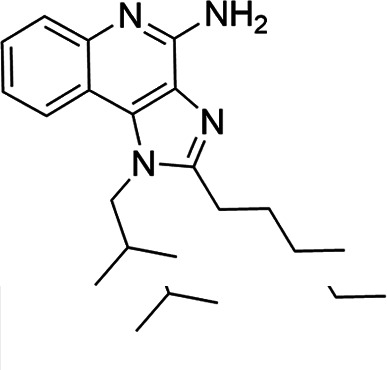	3.2 ± 0.06	15 ± 0.03	3.4 ± 0.79	5.4 ± 0.79	9.54 ± 0.6	2.8	1.76	7.54 ± 0.6	2.21	1.36
2	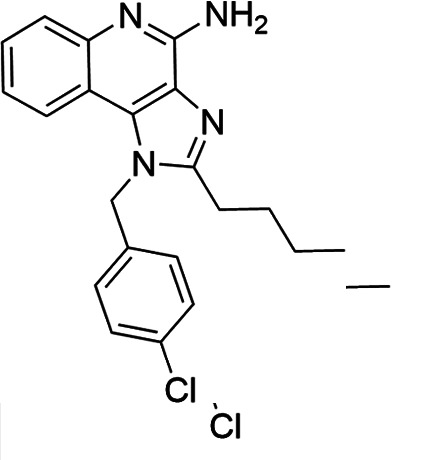	2.92 ± 0.06	n/a	3.57 ± 0.58	5.6 ± 0.62	5.76 ± 0.6	1.61	1.02	3.86 ± 0.6	1.08	0.68
3	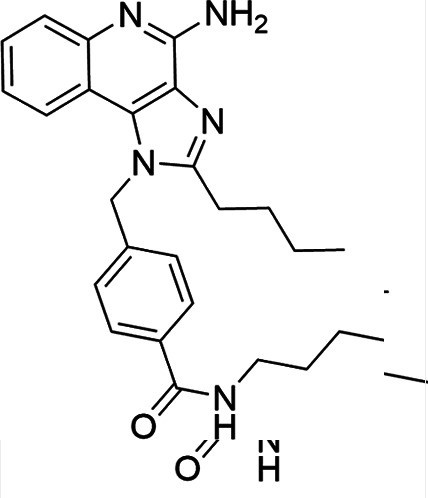	0.35 ± 0.06	n/a	5.8 ± 0.4	7.86 ± 0.7	10.9 ± 0.2	1.87	1.38	7.46 ± 0.5	1.28	0.94
4	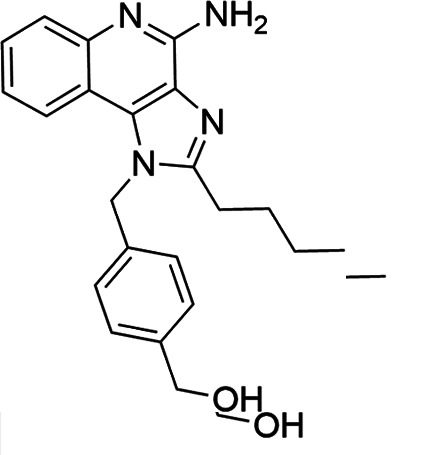	0.22 ± 0.02	n/a	6.5 ± 0.4	7.4 ± 0.52	6.75 ± 0.2	1.03	0.91	4.75 ± 0.2	0.73	0.64
5	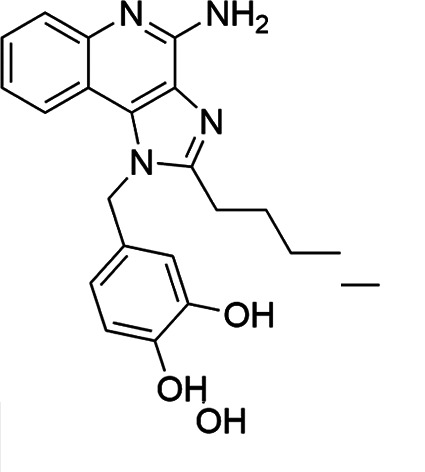	0.15 ± 0.06	2.75 ± 0.07	2.28 ± 0.62	3.29 ± 0.61	34.4 ± 0.5	15.08	10.45	32.5 ± 0.5	14.25	9.87
6	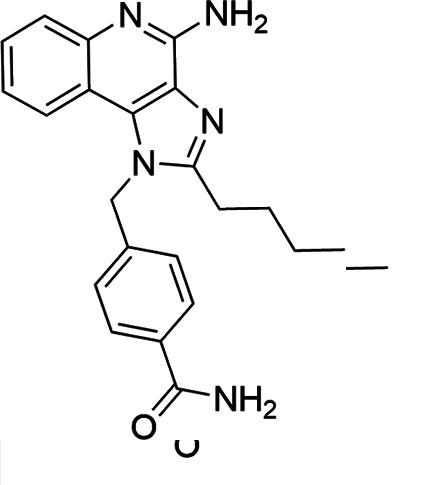	0.32 ± 0.03	18.25 ± 0.43	5.1 ± 0.26	7.06 ± 0.3	6.7 ± 0.4	1.31	0.94	4.55 ± 0.5	0.89	0.64
7	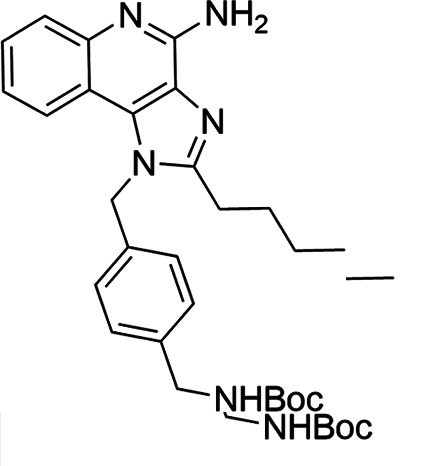	Active	Active	3.82 ± 0.39	5.16 ± 0.79	6.28 ± 0.2	1.64	1.21	4.28 ± 0.2	1.12	0.86
8	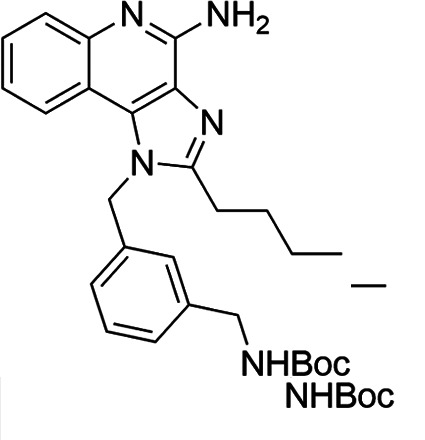	Active	Active	2.21 ± 0.6	3.1 ± 0.9	9.7 ± 1	4.38	3.12	8.86 ± 0.5	2.02	2.83
9	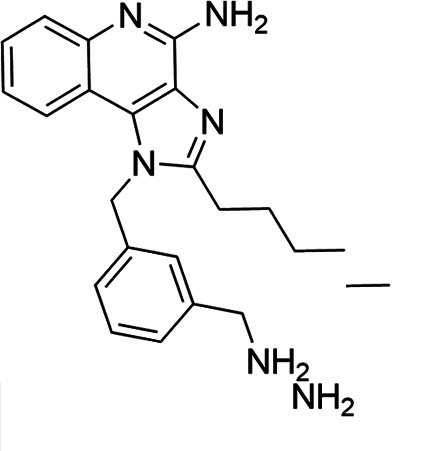	0.11 ± 0.032	1.70 ± 0.054	4.53 ± 0.58	6.46 ± 0.47	6.67 ± 0.3	1.47	1.03	4.67 ± 0.3	1.03	0.7
10	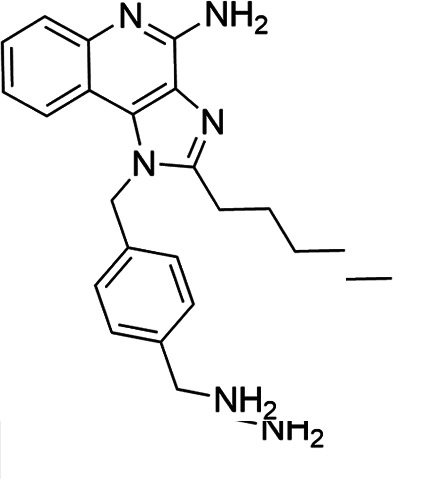	0.027 ± 0.008	1.032 ± 0.018	2.33 ± 0.4	3.0 ± 0.36	30.37 ± 1.6	13.034	10.12	29.37 ± 1.6	12.6	9.79
11	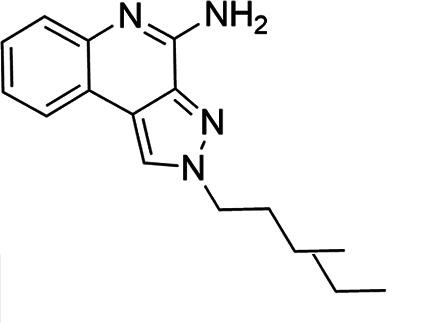	0.19	0.056	4.63 ± 0.45	6.6 ± 0.4	6.67 ± 0.2	1.44	1.01	4.67 ± 0.2	1	0.7
Resiquimod	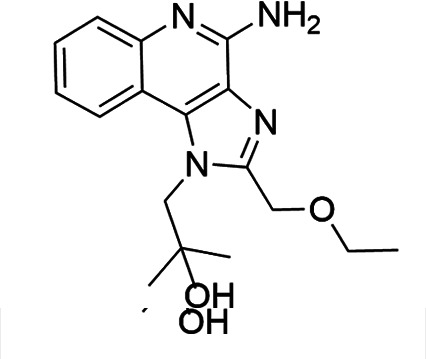	0.31 ± 0.02	3.5 ± 0.09	3.46 ± 0.1	4.46 ± 0.66	11.46 ± 0.5	3.31	2.56	9.0 ± 0.6	2.64	2.01
Amphotericin B				0.13 ± 0.06	0.2 ± 0.1	19.15 ± 1.0	147.3	95.75	16.18 ± 0.7	161	80.5
Miltefosine				2.43 ± 0.77	3.43 ± 0.77	26.0 ± 1.0	10.69	7.58	20.1 ± 0.9	8.27	5.86

^*a*^ IC_50_ values represent the concentration (μg/mL) required to inhibit 50% of *Leishmania* promastigotes or intracellular amastigotes. CC_50_ indicates the concentration (μg/mL) that causes 50% cytotoxicity in RAW 264.7 macrophages or HeLa cells, as determined by the MTT assay. Values are presented as mean ± standard deviation from three independent experiments. Selectivity index(SI) was calculated as the ratio of CC_50_ to IC_50_ for both promastigote and amastigote stages, indicating compound selectivity toward parasites over host cells. AmB and MF were included as standard antileishmanial drugs; Resiquimod was used as a reference TLR7/8 agonist. n/a, not available.

### Intracellular ROS production as a marker of antileishmanial activity

**Fig 5:**
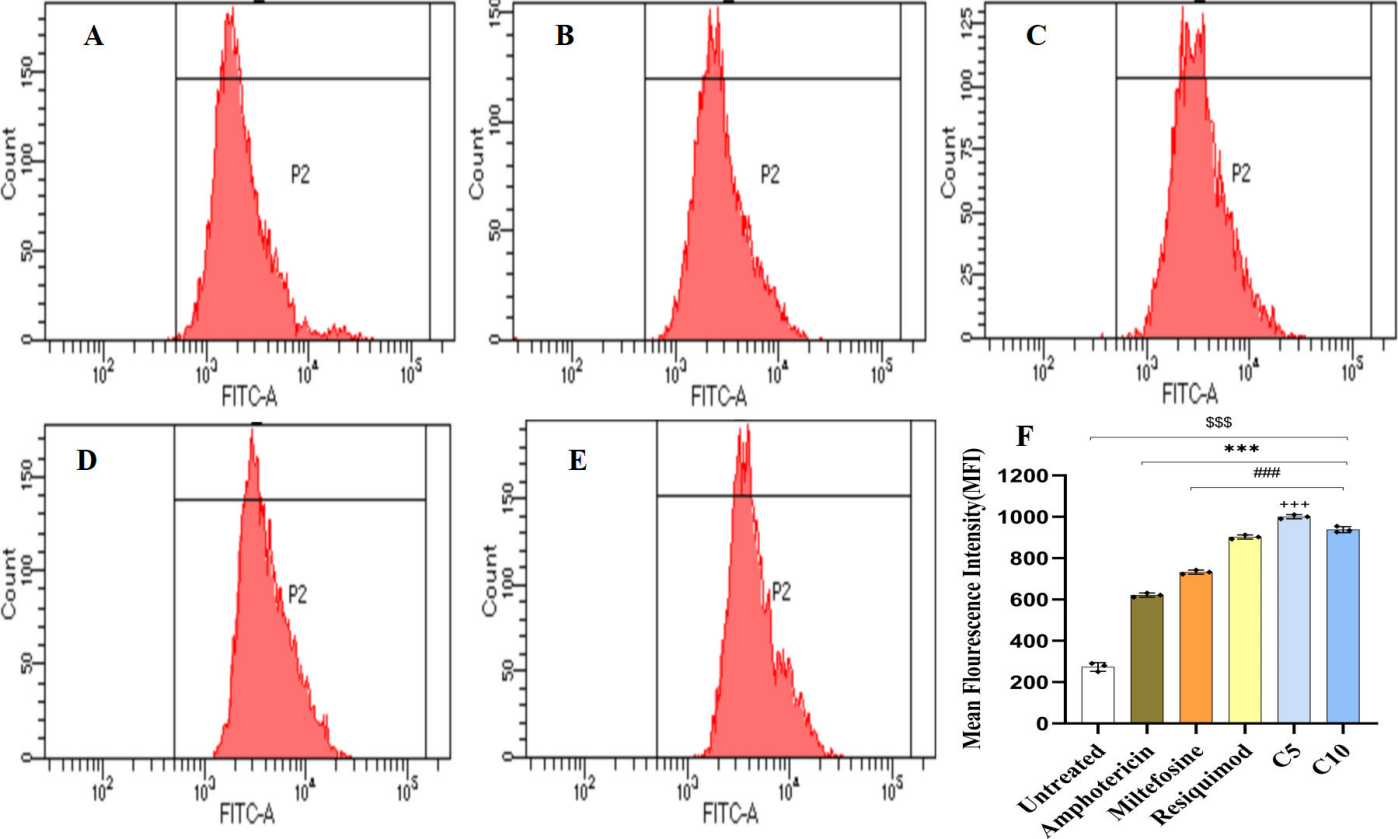
Intracellular ROS production by promastigotes of *L. donovani* after incubation with (**A**) AmB, (**B**) MF, (**C**) Resiquimod, (**D**) compound 10, (**E**) compound 5, (**F**) histogram of P2 gated population and bar graphs depicting mean fluorescence intensity (MFI). ROS levels were measured using 2′,7′-dichlorofluorescin diacetate (DCF-DA) fluorescence and analyzed by flow cytometry. Results are expressed as MFI. MFI represented as fluorescein isothiocyanate (FITC) mean: the MFI values depicted in the figure correspond to the mean fluorescence intensity of cells stained with a fluorochrome conjugated to FITC. Data represent mean ± standard deviation from three independent experiments (^$$$^*P* < 0.0001, untreated vs AmB, MF, Resiquimod, compound 10, and compound 5; ***P < 0.0001, AmB vs MF, Resiquimod, and compound 10; ^###^*P* < 0.0001, MF vs Resiquimod, compound 10, and compound 5; ^+++^*P* < 0.0001, Resiquimod vs compound 5). The statistical significance was determined using one-way analysis of variance (Tukey’s multiple comparison test).

Out of the 11 TLR7/8 agonists, 2 hit compounds (5 and 10) with the highest SI values were selected for further evaluation to elucidate their importance in elevating oxidative stress in promastigotes. The results revealed a significant increase in ROS production in parasites incubated with both compound 5 and compound 10 in comparison to untreated controls (mean fluorescence intensity [MFI] of 295 ± 1.52, 95% confidence interval [CI]: 292.15–297.85). The MFIs of compounds 5 and 10 were found to be 1,002.0 ± 1.52 (95% CI: 999.15–1,004.85) and 935 ± 1 (95% CI: 933.06–936.94), respectively, which were significantly higher than all the standards, i.e., AmB, MF, and Resiquimod (*P* < 0.001, one-way analysis of variance with Tukey’s post hoc test). Resiquimod had its MFI significantly higher (457 ± 1.9, 95% CI: 454.09–459.91, *P* < 0.001) than AmB and MF, which indicated the potential of TLR7/8 in killing the intracellular parasite by inducing the promastigotes to produce higher ROS in comparison to currently available drugs (AmB and MF) (Fig. 5). These findings indicate that the selected TLR7/8 agonists enhance intracellular oxidative stress in *L. donovani* promastigotes to a greater extent than current therapeutics (AmB and MF).

### TLR7/8 agonist-induced nitric oxide production in macrophages enhances antileishmanial host defense

**Fig 6:**
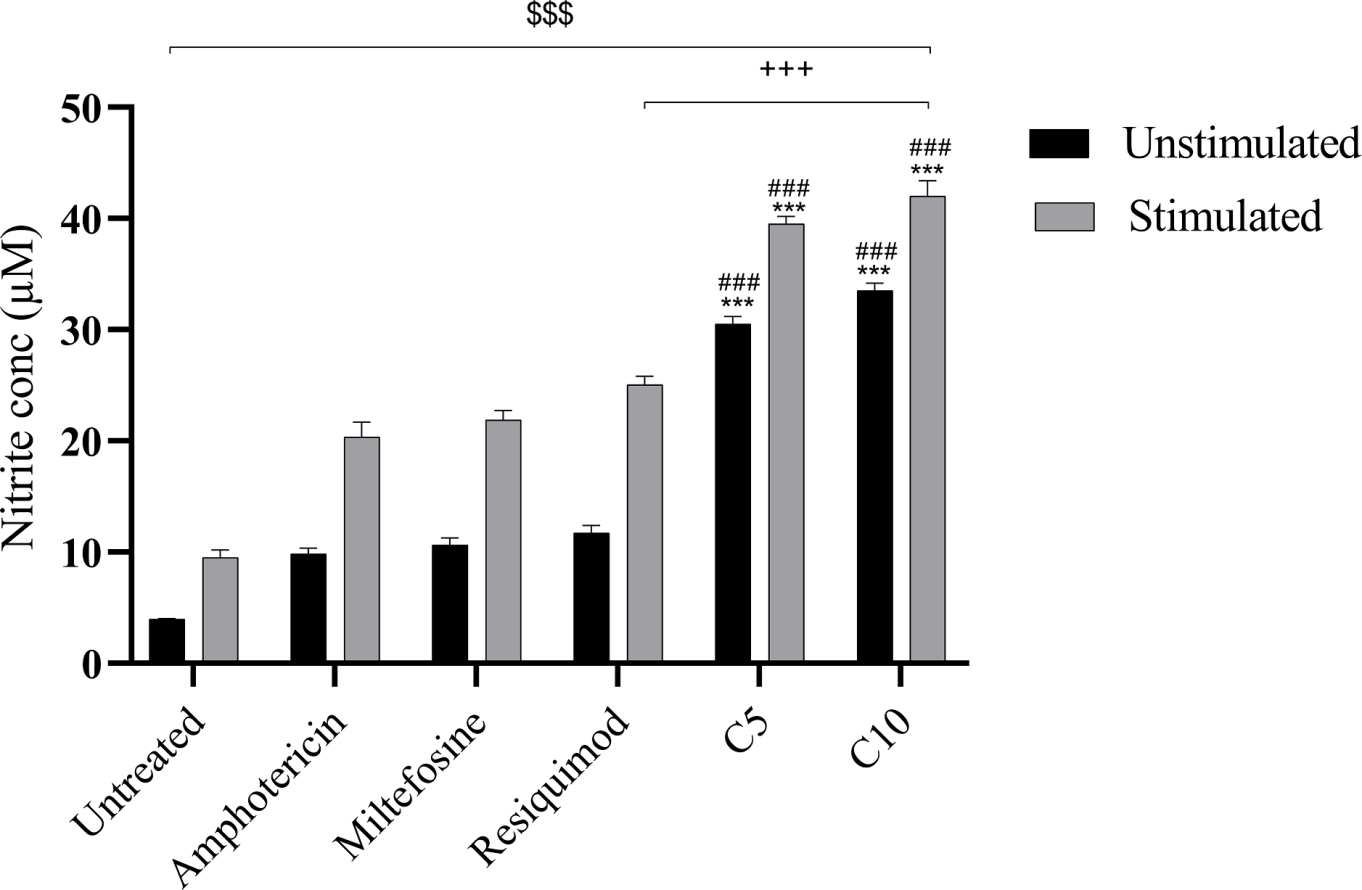
Induction of nitric oxide in RAW macrophages after incubation with compound 5 (C5), compound 10 (C10), Resiquimod, and standard antileishmanial drugs using Griess reagent. Data represent mean ± standard deviation from three independent experiments (^$$$^*P* < 0.0001, untreated vs AmB, MF, resiquimod, compound 5, and compound 10; ***P < 0.0001, AmB vs Resiquimod, compound 5, and compound 10; ^###^*P* < 0.0001, MF vs compound 5 and compound 10; ^+++^*P* < 0.0001, Resiquimod vs compound 5). The statistical significance was determined using one-way analysis of variance (Tukey’s multiple comparison test).

NO is a crucial aspect in macrophage-mediated immunity, specifically in host defense against *L. donovani*. To evaluate the immunomodulatory capacity of selected TLR7/8 agonists, NO production was quantified in RAW 264.7 macrophages following 48 hour treatment with compounds 5 and 10, along with reference drugs AmB, MF, and Resiquimod (Fig. 6). The results depicted a significant elevation in NO levels in all treated cells in comparison to the untreated one. Compound 10 showed the highest NO production, i.e., 42 ± 1 µM followed by compound 5, i.e., 39 ± 1 µM, which was significantly higher in comparison to all the standard groups. Treatment with AmB and MF resulted in a moderate increase in NO levels, which were 20.3 ± 0.8 µM and 21.9 ± 0.6 µM, while resiquimod resulted in higher upregulation of NO, i.e., 25.0 ± 0.8 µM. The enhanced NO production induced by compounds 5 and 10 strongly correlates with macrophage activation and highlights their potential as immunomodulatory agents capable of stimulating host innate responses against *L. donovani*.

### Impact of TLR7/8 agonist on cell cycle progression in *Leishmania donovani*

**Fig 7:**
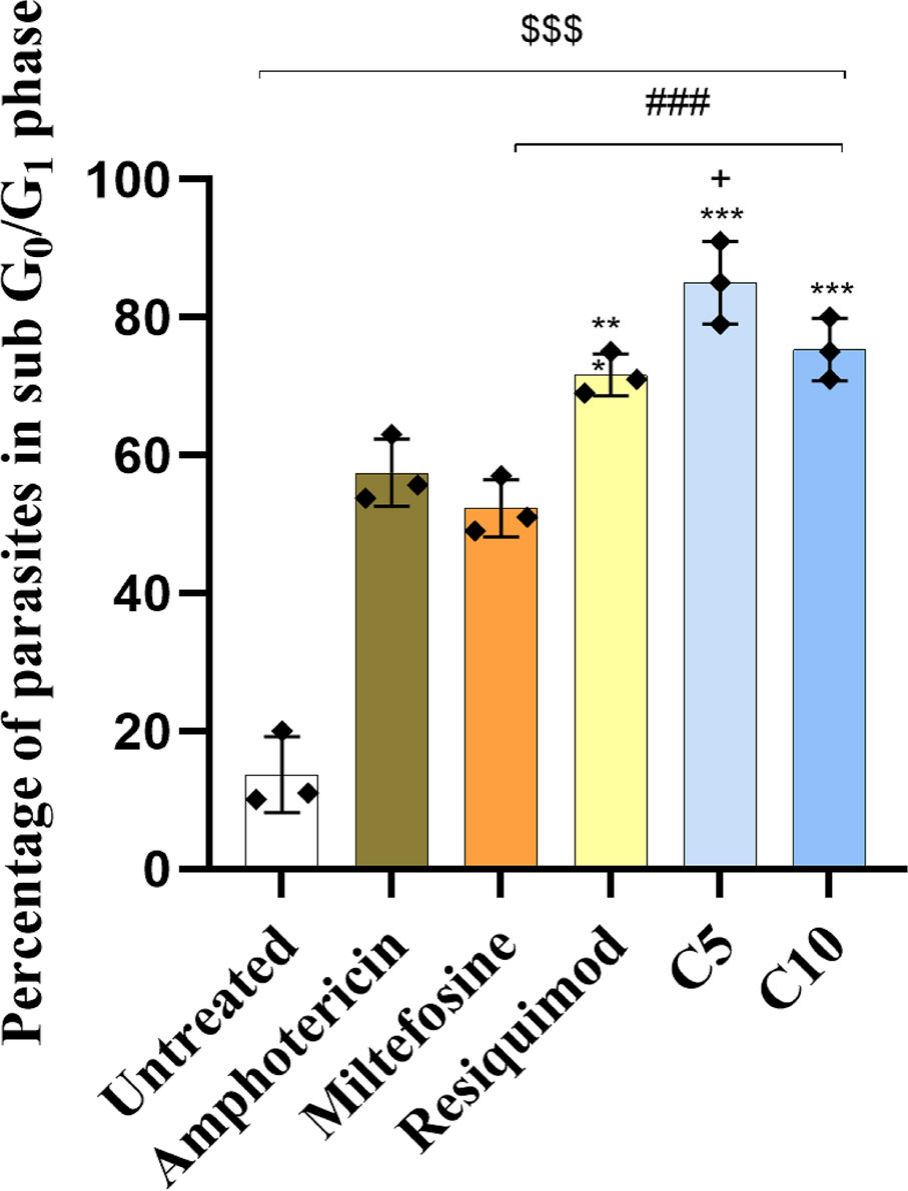
Promastigotes were treated with AmB, MF, Resiquimod, compound 5, and 10 (C5 and C10). Bar graphs depict the percentage of parasites in the sub-G_0_/G_1_ phase, indicative of apoptotic cell populations, as determined by flow cytometry. Data represent mean ± standard deviation from three independent experiments (^$$$^*P* < 0.0001, untreated vs AmB, MF, Resiquimod, compound 5, and compound 10; ***P < 0.0001, AmB vs Resiquimod, compound 5, and compound 10; ^###^*P* < 0.0001, MF vs Resiquimod, compound 5, and compound 10; ^+^*P* < 0.0001, Resiquimod vs compound 5. The statistical significance was determined using one-way analysis of variance (Tukey’s multiple comparison test).

Cell cycle analysis in *L. donovani* is an important aspect for interpreting the mechanism of action of TLR7/8 agonists. The induction of G_0_/G_1_ arrest indicates the suppression of parasite multiplication (Fig. 7). Thus, PI staining through flow cytometry was used to ascertain the effect of TLR7/8 agonist on cell cycle progression. Untreated parasites exhibited normal cell cycle distribution with only 12.0% ± 1.05% parasites in sub G_0_/G_1_ phase. Treatment with AmB and MF resulted in a marked increase to 57.0% and 52.3% in comparison to untreated (*P* < 0.001). Resiquimod treatment further elevated this percentage to 71%, which was significantly higher than MF (*P* < 0.01) and AmB (*P* < 0.001), suggesting enhanced apoptotic activity. However, treatment with compound 5 and compound 10 resulted in significantly higher (*P* < 0.001) arrest in the sub-G_0_/G_1_ phase, i.e., 80% ± 1.2% and 75.7% ± 1.25% in comparison to standard drugs AmB and MF. In comparison to Resiquimod, also the percentage of parasites in the sub-G_0_/G_1_ phase in compound 5 treatment was significantly higher (*P* < 0.05). These findings suggest that compounds 5 and 10 may exert antiparasitic effects through enhanced induction of apoptotic cell death, possibly via innate immune modulation and direct cytotoxic activity.

## DISCUSSION

Visceral leishmaniasis remains a major health challenge in endemic regions due to drug resistance, high costs, and toxicity of current treatments (26). Despite ongoing drug development efforts, there remains a limited knowledge of agents that can simultaneously target *Leishmania* parasites and activate host immune responses. Developing new agents using known antileishmanial scaffolds like quinolines is a key strategy (27). TLRs, which recognize microbial PAMPs and activate innate immunity, are promising targets. Notably, although TLR7/8 agonists have been extensively investigated in antiviral, oncologic, and certain parasitic disease contexts, their integrated role as both immunomodulators and direct antileishmanial agents against *L. donovani* has remained largely unexplored. The present study addresses this critical gap by establishing the dual-target paradigm as a viable strategy for VL. In doing so, our findings advance the antileishmanial drug discovery pipeline by proposing a mechanistically distinct therapeutic class that not only potentiates host-directed immune responses but also exerts direct parasiticidal effects. This approach represents a departure from existing VL therapies, which predominantly act via parasite-specific biochemical pathways and opens avenues for rational design of next-generation agents with improved efficacy and reducing emergence of resistance. In this study, the antileishmanial efficacy of TLR7/8 agonists was evaluated against *L. donovani*. IC_50_ values of the selected TLR7/8 agonists ranged between 2.28 ± 0.62 µg/mL and 6.5 ± 0.4 µg/mL. Results depicted that although all compounds had good antileishmanial activity, compound 5 and compound 10 were the most effective, showing the lowest IC_50_ values. By assessing IC_50_ against promastigotes, this objective addresses the knowledge gap of whether TLR7/8 agonists have inherent direct parasiticidal effects, beyond immunomodulation, highlighting their novelty as dual-acting compounds. This aligns with their previously reported low EC_50_ values for TLR7 (0.027 µM for compound 5 and 0.15 µM for compound 10) and TLR8 (1.03 µM for compound 5 and 2.75 µM for compound 10) (17, 18). Similar kinds of studies on imidazoquinoline have shown a synergistic antileishmanial effect between morphine and imiquimod against *Leishmania infantum* with IC_50_ values 0.102 ± 0.03 µM for morphine, 0.235 ± 0.01 µM for imiquimod, and 0.173 ±  0.02 µM for the combination (12). Additionally, investigations on imidazo[1,2*a*]pyrimidine derivatives showed promising antileishmanial potential, and the compound demonstrated an IC_50_ value of 6.63 µM against promastigote (28). These comparative values highlight that while other chemotypes show activity, they often lack the combined immune-activating capacity demonstrated here, setting this study apart in its translational scope.

Susceptibility of promastigotes toward the compound differs from amastigotes residing inside the cell (29). IC_50_ values of TLR7/8 agonists ranged from 3.29 ± 0.61 µg/mL to 7.8 ± 0.7 µg/mL, with compounds 5 and 10 showing the best activity. Measuring antiamastigote IC_50_ addresses the gap of evaluating intracellular efficacy, confirming that these compounds not only target extracellular parasites but also activate macrophage-mediated killing, supporting their immunomodulatory novelty. A similar kind of antiamastigote study depicted antileishmanial activity of β-acetyl-digitoxin against *L. infantum*, with IC_50_ values of 20.94 ± 2.60 µM for β-acetyl-digitoxin and 0.11 ± 0.03 µM for AmB (30). Another study revealed IC_50_ values of 1.81 µg/mL for MF and 0.09 µg/mL for resveratrol against *Leishmania* in peritoneal macrophages (31). By demonstrating intracellular efficacy in macrophages, these results directly enlighten lead optimization criteria for drug candidates in the VL pipeline, where macrophage-resident parasites represent the main therapeutic target.

The CC_50_ evaluation was performed in RAW 264.7 macrophages and HeLa cell line to assess the cytotoxicity of TLR7/8 agonists. RAW cells were chosen since they are primary host cells for *Leishmania*, making them key targets for intervention (32). On the contrary, HeLa cells gave an insight into broader cytotoxicity profile in non-immune and non-phagocytic cell type (33). By including HeLa cells, this experiment directly tackles the existing gap of general safety, demonstrating that TLR7/8 agonists are selectively active against *L. donovani* while minimally affecting non-immune cells, emphasizing translational novelty. The CC_50_ values were lower in HeLa cells in comparison to RAW macrophages, implying a bit higher sensitivity of epithelial cells toward TLR7/8 agonists. In both cell lines, the highest CC_50_ values were observed in compounds 5 and 10. In a similar study, cytotoxic evaluation of TLR7/8 agonists was done on peritoneal macrophages, and the CC_50_ values were 11.25 and 16.67 µM (9). Additionally, research was carried out with troponoids in epithelial cell line HepDES19, where CC_50_ values for all 38 compounds ranged from 17 to >100 µM (34). The selective cytotoxicity profile observed here strengthens the case for these molecules entering preclinical development, where off-target toxicity is a major cause of attribution in antiparasitic drug pipelines.

SI, calculated as CC_50_/IC_50_, was higher in RAW 264.7 macrophages, indicating targeted antiparasitic action and the ability of compounds to activate innate immune cells for parasite clearance (35). For both the cell lines and forms of parasites, compounds 5 and 10 had better SI and were carried forward for further immunomodulatory and parasiticidal evaluations such as ROS and NO generation and cell cycle analysis. A study using a similar kind of compounds was performed, and SI values of 0.99 and 2.81 were achieved (9). Another study evaluated SI by taking the ratio of CC_50_ on KB cells and IC_50_ on amastigotes using a series of 9 quinolines and 18 styrylquinoline-based compounds, and the top compound had an SI value of 121.5 (36).

Exorbitant ROS can disintegrate cellular homeostasis, resulting in oxidative damage of protein, lipids, and DNA, eventually leading to promastigote death (37). This approach fills the current void in knowledge by understanding the direct oxidative stress-mediated parasiticidal mechanism, demonstrating the novelty of dual action: both immunomodulatory (via macrophage) and direct parasite killing. Our data indicate that compounds 5 and 10 induce elevated levels of ROS within promastigotes, leading to oxidative stress-mediated death, similar to previously reported antileishmanial compounds such as 4,7-dihydroxyflavone (24). While amphotericin B and miltefosine act primarily through lipid membrane disruption, our results suggest that TLR7/8 agonists may exert additional direct parasiticidal effects independent of their immunomodulatory activity in host cells. These mechanistic insights broaden the understanding of TLR-based compounds in parasitic diseases, providing a template for rational combination therapies that could exploit oxidative stress pathways.

NO estimation in RAW macrophages treated with TLR7/8 agonists, with or without LPS stimulation, highlighted their immunomodulatory potential. Demonstrating NO induction, this study fills the knowledge gap regarding the ability of these compounds to activate macrophage effector functions, underscoring their novelty as potential agents for host-directed parasite clearance (38). In unstimulated macrophages, compounds 5 and 10 induced moderate NO production, indicating intrinsic immunostimulatory activity. With LPS stimulation, TLR7/8 agonists significantly elevated NO levels compared to AmB and MF, supporting their role as immunostimulants and potential adjuvants in antileishmanial vaccines. In a related investigation, the ability of TLR agonists in enhancing NO levels both in the presence or absence of stimuli was observed (39). This immunostimulatory dimension is particularly relevant for vaccine adjuvant development in VL, where generating a sustained macrophage response is a major challenge.

Cell cycle analysis reveals how TLR7/8 agonists act by depicting whether they block parasite replication by arresting the G_0_/G_1_ or S phase. This is specifically important for the promastigote stage, since inhibition of their proliferation can limit parasite load (40). This objective addresses the missing link between parasite growth inhibition and immune activation, demonstrating the dual novelty of TLR7/TLR8 agonists as both direct parasiticidal agents and potent immune activators. Treatment of promastigotes with compounds 5 and 10 resulted in a significant increase in the percentage of parasite arrest in the G_0_/G_1_ phase. The observed G_0_/G_1_ arrest suggests that the compound interferes at a critical checkpoint in parasite replication, effectively preventing DNA synthesis. This aligns with earlier reports that G_1_ is a key regulatory stage in *Leishmania* cell cycle progression (41). In a study where cell cycle analysis was done using the same protocol, curcumin was used at 80 µM concentration, and 82.5% of cell death was achieved (42).

**Fig 8:**
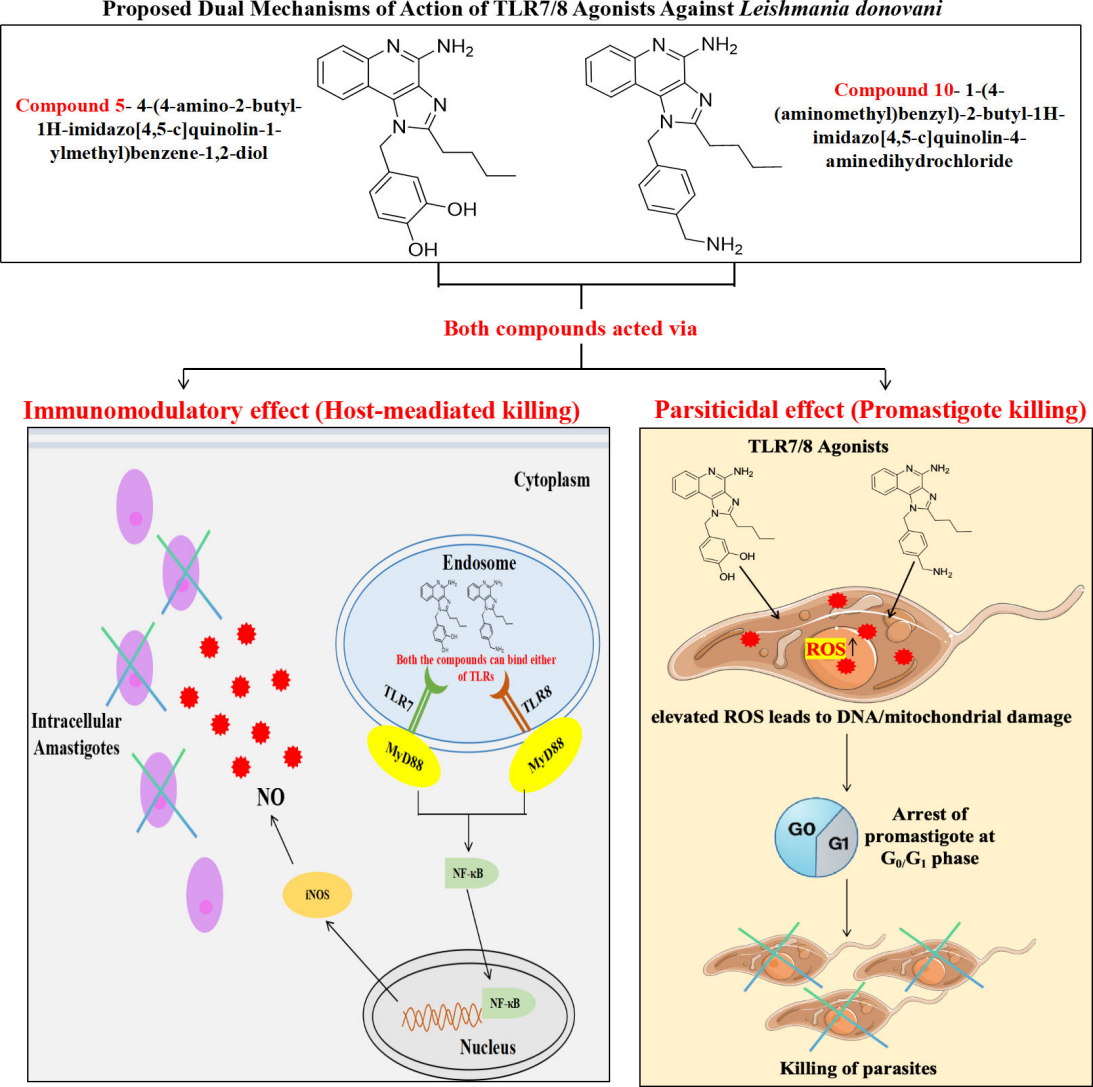
Experimental workflow showing dual action of synthetic TLR7/8 agonists against *Leishmania donovani*: immunomodulation via nuclear factor kappa-B/inducible nitric oxide synthase–NO pathway and direct parasiticidal effects through ROS-induced cell cycle arrest and parasite death.

Thus, considering the abovementioned observations, TLR7/8 agonists showed strong antileishmanial activity with low IC_50_ values, good cytotoxicity profiles, ROS induction in promastigotes, increased NO in macrophages, and disrupted parasite replication. Each of these measured objectives collectively filled the knowledge gap of identifying compounds that combine direct parasiticidal action with immunomodulation, explicitly highlighting their novelty as dual-acting therapeutic agents. Importantly, the integration of these findings offers a multi-layered preclinical profile covering efficacy, safety, and mechanism, which positions these TLR7/8 agonists as credible candidates in the antileishmanial drug discovery pipeline. To better illustrate the dual immunomodulatory and parasiticidal mechanisms proposed for compounds 5 and 10, a schematic model is presented (Fig. 8).

In summary, this study is the first to provide an integrated evaluation of selective TLR7/8 agonists for VL, encompassing cellular, biochemical, and immunological perspectives. From a cellular standpoint, the study assessed parasite viability (IC_50_ against promastigotes and intracellular amastigotes), host cytotoxicity (CC_50_ in RAW macrophages and HeLa cells), and cell cycle disruption (G_0_/G_1_ arrest in *L. donovani*), offering insights into the direct leishmanicidal activity and selectivity of the compounds. Biochemically, the induction of ROS in parasites and NO in macrophages highlighted the compounds’ ability to generate oxidative stress, a key mechanism in parasite killing. From an immunological perspective, the study demonstrated activation of macrophage effector functions, both in the presence and absence of LPS stimulation, reflecting the intrinsic immunostimulatory properties of these TLR7/8 agonists. Collectively, these results differentiate the current work from previous studies on TLR agonists in leishmaniasis, which have largely been limited to cutaneous forms and lacked a systematic dual-mechanism analysis. Together, these findings fill the knowledge gap on dual-action therapeutics in VL, clearly establishing the novelty of TLR7/8 agonists as agents that combine direct antiparasitic effects with robust immunomodulation, thereby reinforcing their translational promise as host-directed therapies or vaccine adjuvants. Future work could expand this evaluation to *in vivo* VL models, explore synergistic combinations with existing drugs, and assess the adjuvant potential of these molecules in prophylactic vaccine formulations—steps that will be essential for moving these leads toward clinical application.

### Conclusion

This study presents the first comprehensive biological evaluation of selective TLR7/8 agonists in the context of VL, integrating cellular, biochemical, and immunological approaches. The derivatives, particularly compounds 5 and 10, demonstrated potent antileishmanial activity against both promastigote and intracellular amastigote forms of *L. donovani*, with favorable cytotoxicity profiles in mammalian cell lines. At the cellular level, the compounds induced significant G_0_/G_1_ arrest in parasites, indicating disruption of parasite proliferation. Biochemically, both compounds effectively elevated ROS in parasites and NO levels in macrophages, pointing to oxidative damage as a mechanism of parasite killing. Immunologically, they enhanced macrophage activation, even in the absence of LPS stimulation, highlighting their intrinsic immune-stimulatory capacity.

Taken together, this integrated analysis establishes TLR7/8 agonists as dual-function agents directly targeting *Leishmania* parasites while simultaneously activating innate immune mechanisms. Unlike previous reports which focused primarily on cutaneous leishmaniasis, this study delineates their potential for VL and provides a foundational framework for their future use as both therapeutic candidates and vaccine adjuvants in host-directed strategies against VL.
